# Ecological Features and Conservation of *Urtica rupestris* Guss. (Urticaceae): A Narrow Endemic Species of Sicily

**DOI:** 10.3390/plants12010164

**Published:** 2022-12-29

**Authors:** Saverio Sciandrello, Salvatore Cambria, Gianpietro Giusso del Galdo, Pietro Minissale, Marta Puglisi, Gianmarco Tavilla, Valeria Tomaselli

**Affiliations:** 1 Department of Biological, Geological and Environmental Sciences, University of Catania, Via A. Longo 19, 95125 Catania, Italy; 2 Department of Biosciences, Biotechnologies and Environment, University of Bari “Aldo Moro”, Via Orabona 4, 70125 Bari, Italy

**Keywords:** *Adiantetea capilli-veneris*, conservation, demographic analysis, ecology, IUCN, plant conservation, phytosociology, rupicolous habitat

## Abstract

The conservation actions of endangered plant species require a clear knowledge of their habitats. *Urtica rupestris* Guss. (Urticaceae) is a rare endemic plant species occurring on shady cliffs in the southern-eastern part of Sicily. In the last century, the extreme anthropogenic alterations of Hyblaean plateau have caused the continuous and unrestrained fragmentation of natural habitats and consequently the reduction and disappearance of some plant species. A total of 52 vegetation plots, of which 34 are unpublished, were analyzed in order to characterize the floristic composition of the *U. rupestris* community. All the relevés were classified using classification and ordination methods. The species is mainly linked to shady and wet rock habitats, and only secondarily colonizes the undergrowth shrubs. According to IUCN criteria, we propose a new risk status for this species and the establishment of a new habitat (92/43CEE) for correct long-term conservation. Finally, a new association, *Urtico rupestris-Adiantetum capilli-veneris*, which falls within the *Polysticho setiferi-Phyllitidion scolopendrii* alliance (*Adiantetea capilli-veneris* class), was described. This study can provide useful information for the management and conservation of *U. rupestris*.

## 1. Introduction

Defining effective plant conservation strategies has become a crucial issue in the Mediterranean area due to strong human pressure on the natural landscape, which has led to the loss of habitats and some endemic plant species [1,2]. Sicily is one of the most important centers of plant diversity in the Mediterranean region, with many habitats included in Annex I of the Habitat Directive [3,4,5,6,7,8,9]. The importance of the vascular flora of Sicily lies not only in the total number of taxa (2763 species according to Bartolucci et al. [10]), but also in the considerable number of endemic species (400 species according to Peruzzi et al. [11]). In particular, the south-eastern sector of the island (Hyblaean territory), despite relatively low altitudes, hosts an extraordinary floristic richness and several plant communities, as well as many habitat types [12,13,14]. This high biological variety is due to the great geological, geomorphological, and bioclimatic complexity of the Hyblaean territory. In this area, several rare and narrow endemic species grow, such as *Zelkova sicula* Di Pasq., Garfì & Quézel, *Trachelium caeruleum* L. subsp. *lanceolatum* (Guss.) Arcang., *Anthemis pignattiorum* Guarino, Raimondo & Domina, *Limonium syracusanum* Brullo, *L. pachynense* Brullo, *L. pavonianum* Brullo, *Ferulago nodosa* (L.) Boiss. subsp. *geniculata* (Guss.) Troìa & Raimondo, *Myosotis tineoi* C.Brullo & Brullo, *Epipactis hyblaea* Brullo & Zimmitti, and *Solenopsis laurentia* (L.) C.Presl. subsp. *hyblaea* Brullo et al. [15,16,17]. In the list of endemic species of the Hyblean territory, there is also *Urtica rupestris*, which shows some affinities (*morifolia*-clade) with *U. morifolia* Poir. (Macaronesian Islands) and a strong similarity with *U. fragilis* J.Thiébaut (Syria, SE Turkey, Lebanon) [18]. *Urtica rupestris*, exclusive to the southern-eastern part of Sicily, is an Urticaceae woody nettle species that grows on the shady cliffs of the Hyblaean district. It was listed in the Red Book of Italian flora [19,20] as a vulnerable species (VU). The aim of our research was to study the scattered surviving *U. rupestris* population, in particular, analyzing the floristic composition, identifying the ecological requirements, and re-evaluating the conservation status at a regional level. Moreover, we propose the establishment of a new habitat type according to the European Directive 92/43CEE in order to achieve long-term conservation of the species.

## 2. Results and Discussion

### 2.1. Description of the Species (Based on our Specimens Collected in Monello and Palombara Localities)

Based on our morphological investigations of *Urtica rupestris*, we report below an updated and more detailed description of the species than those available in the literature.

*Urtica rupestris* Guss., Cat. Pl. Hort. Boccadifalco: 83(–84). 1821. Type (lectotype designated by Corsi et al. [21]: 218) “*Militello di Val di Noto nel vallone detto il Carcarone. Ad rupes vulcanicas in umbrosis vallibus*, *Aprili. Majo*”. (Herb. Guss. NAP).

The species is an erect, perennial, rhizomatous herb 0.3–0.8(−1.0 m) that is woody at the base and forms a perennial root with many unbranched stems. The plants are mostly dioecious and sometimes monoecious. The plant has a very sparse cover of erect stinging hairs that are 0.9–1.2 mm long, with a pluricellular straight base and a ca. 1/3–1/2 of the overall length of the seta. It is subglabrous with scattered, simple trichomes that are 0.1–0.4 mm long. The leaf lamina are 50–100 × 30–50 Mm ovate-acuminate, cuneate, or truncate at the base. The surface is poorly pubescent with short simple trichomes that are 0.1–0.5 mm long and has very few stinging hairs. The margins are coarsely and regularly serrate, with 8–9 teeth on each side. These teeth are 5–7 mm long and are usually undivided. The leaves are opposite and are deciduous with an apex acute to acuminate. The stipules are free (4 per node) and 3–4 mm long. The petioles are 25–45 mm long. The leaves are thin, dark green, and shiny on the upper side and lighter green on the lower leaf page. The racemes are unisexual. The plant has staminate flowers with tepals that are 0.6–0.8 mm long and pistillate flowers with tepals that are 1–1.2 mm long, which are sparsely pubescent. The female inflorescence is 8–18 mm long and is shorter than the subtending petiole, patent, or pendent in fruit. The male inflorescence is 20–40 mm long and is erecto-patent. The female flowers have subglabrous perianth segments. The mature fruit has tepals of 1.2–1.3 mm long and are achenes ovoid, wrinkled, and 1.2–1.3 × 0.8–0.9 mm.

Chromosome number: 2n = 26 according to Corsi et al. [21].

### 2.2. Ecological Data and Habitat Analysis

**Figure 1:**
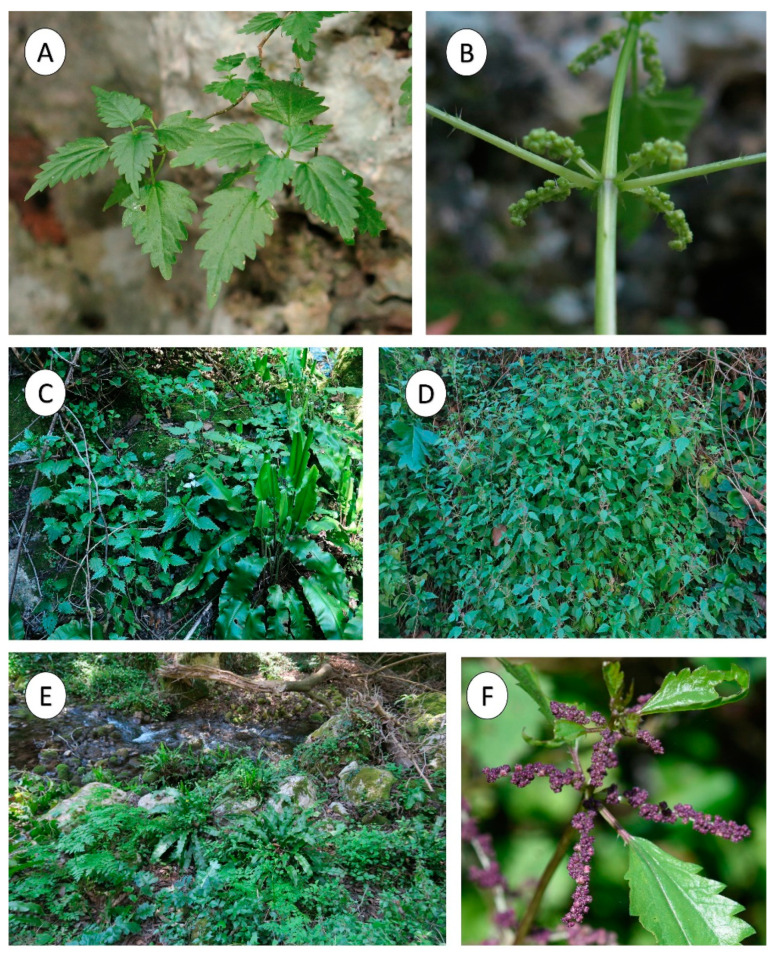
Some views of *Urtica rupestris.* (**A**) On a limestone cliff; (**B**) Female inflorescence; (**C**) *Urtica rupestris* with *Asplenium scolopendrium* from Cava Bibbinello (Syracuse); (**D**) Undergrowth plant from Anapo valley (Syracuse); (**E**) *Urtico rupestris-Adiantetum capilli-veneris* from Cava Bibbinello (Syracuse); (**F**) Male inflorescence.

*Urtica rupestris* is a hemicryptophyte scapose, is rhizomatous, and flowers between April and June. It grows on shady cliffs exclusively in the Hyblaean district (south-eastern Sicily), mainly on carbonate rocks and sporadically on volcanic outcrops (Figure 1), although the specimens used to describe the plant species were collected on the volcanic substrates of Calcarone valley near Militello (Type: *Militello di Val di Noto nel vallone detto il Carcarone. Ad rupes vulcanicas in umbrosis vallibus*, *Aprili. Majo*) [21]. The species thrives within valleys that mainly comprise evergreen woodlands dominated by *Quercus ilex* L., of fluvial tectonic origin, with steep slopes. They are locally named “Cave”. It is a member of the highly specialized shady rupicolous community that is rich in bryophytes and pteridophytes and grows in the water dripping crevices of calcareous rocks. It only secondarily colonizes the *Rubus ulmifolius* shrubs (*Scutellario-Urticetum rupestris* Brullo, Minissale, Scelsi, and Spampinato 1993), and in the presence of rock outcrops, also the *Quercus ilex* woods (*Ostryo-Quercetum ilicis* Lapraz 1975, *Doronico-Quercetum ilicis* Barbagallo, Brullo, & Fagotto 1979, *Pistacio-Quercetum ilicis* Brullo & Marcenò 1985), as well as the rare *Laurus nobilis* communities of the *Hedero helicis-Lauretum nobilis* Bueno & Prieto 1991 [22]. Considering the high phytogeographic value of *U. rupestris* and the remarkable naturalistic value of its habitat, as well as its vulnerability, we propose the inclusion of this habitat with the name “Shady wet cliffs (*Adiantetea capilli-veneris*)”, as a new habitat type in Annex I of the Habitat Directive. This shady and wet habitat, with high edaphic humidity, includes the dripping cliffs/walls of the Mediterranean areas that are characterized by chomophytic and chasmophytic vegetation (edaphohygrophylous) related to the *Adiantetea capilli-veneris* class. In the Mediterranean area, this class includes one order and three alliances [23]. In particular, the alliance *Adiantion capilli-veneris* groups plant communities dominated by *Adiantum capillus-veneris* L., which are particularly rich in bryophytes that grow on siliceous or calcareous dripping cliffs. The second one, *Pinguiculion longifoliae*, includes a relict herb-rich chomophytic vegetation of shaded and water-splashed habitats that are dominated by *Pinguicula* L. sp. pl., whereas the *Polysticho setiferi-Phyllitidion scolopendri* groups the fern-rich communities of damp walls and narrow and shady ravines. This shady, wet, and rocky habitat type is characterized by the occurrence of many ferns (*Adiantum capillus-veneris*, *Struthiopteris spicant* (L.) Weiss, *Pteris vittata* L., *Pteris cretica* L., *Osmunda regalis* L., *Asplenium scolopendrium* L. subsp. *scolopendrium*, and *Woodwardia radicans* (L.) Sm.), bryophytes (*Eucladium verticillatum* (Brid.) Bruch & Schimp., *Didymodon tophaceus* (Brid.) Lisa, *Pellia endiviifolia* (Dicks.) Dumort., *Conocephalum conicum* (L.) Dumort., and *Palustriella commutata* (Hedw.) Ochyra), and vascular plants (*Urtica rupestris*, *Cymbalaria pubescens* (C.Presl) Cufod., *Hypericum hircinum* L. subsp. *hircinum*, *Hypericum androsaemum* L., and *Samolus valerandi* L., etc.). As such, this vegetation type is typically found under the Mediterranean macrobioclimate, and occasionally under the sub-Mediterranean variant of the temperate macrobioclimate [24,25]. In Italy, it has been observed in the southern part of the peninsula and in the main islands, as well as in the coastal and sub-coastal areas of the central-northern part of the country [26,27,28,29,30]. These fern-rich plant communities, on thicker and water-rich soils, often come into catenal contact with the phytocoenoses that are dominated by bryophytes of the *Cratoneurion commutati* alliance, including in the Habitat 7220* “Petrifying springs with tufa formation (Cratoneurion)”. The need for a specific habitat type concerning shady dripping cliffs with communities dominated by bryophytes and pteridophytes that belong to the *Adiantetea capilli-veneris* class (shady dripping cliffs with *Woodwardia radicans* and other large ferns) has already been highlighted by Spampinato [31], as well as recently by Guarino et al. [32] and Sciandrello et al. [33]. This proposed Mediterranean dripping cliff habitat includes sciaphilous-hygrophilous communities, in which the density of pteridophytes and bryophytes is high, and rare or endangered ferns of remarkable phytogeographic interest, such as *Woodwardia radicans*, *Osmunda regalis*, *Pteris vittata*, *Pteris cretica*, and *Asplenium scolopendrium* subsp. *scolopendrium*.

### 2.3. Phytosociological Insights

**Figure 2:**
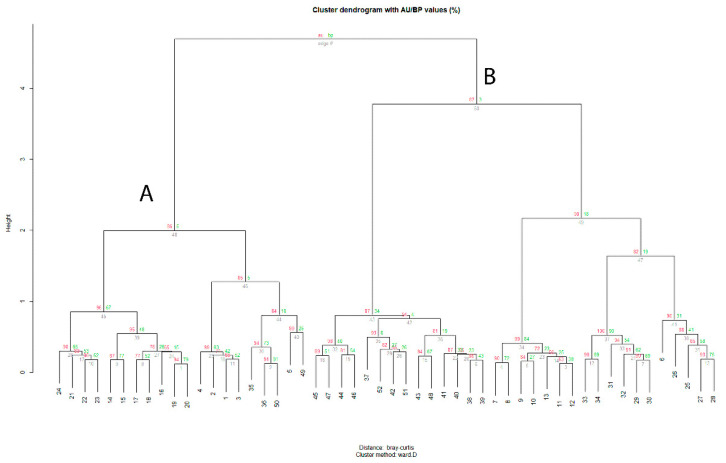
Dendrogram resulting from the cluster analysis (Cophenetic correlation = 0.808) of the surveyed plant communities: (**A**). *Urtico rupestris-Adiantetum capilli-veneris*; (**B**). *Scutellario-Urticetum rupestris*. Approximately unbiased (AU—values printed in red) and bootstrap probability (BP—values printed in green) *p*-values are shown next to the nodes.

**Figure 3:**
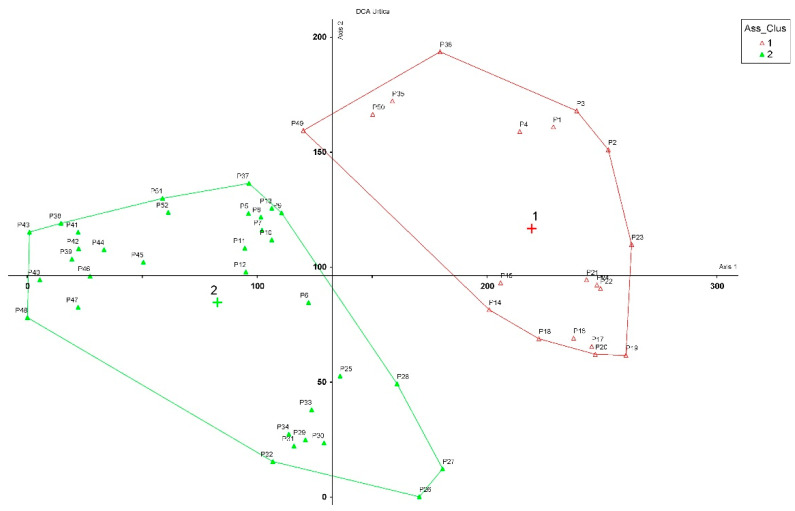
Ordination scatter diagram (DCA). Total variance (‘inertia’) in the species data: 2.1536. The r 2 value of axis 1 is (Eig = 0.44982) and the r 2 value of axis 2 is (Eig = 0.15224). Plant communities: 1. *Urtico rupestris-Adiantetum capilli-veneris*; 2. *Scutellario-Urticetum rupestris*.

The cluster and ordination analysis of all the relevés (52 rel. × 72 sp.) carried out in the Hyblaean territory showed two main groups (Table S1, Appendix A, Figure 2 and Figure 3). The first group (cluster A) includes the shady rupicolous vegetation of the *Adiantetea capilli-veneris* class (20rel. × 46sp.), whereas the second (cluster B) group (32rel. × 56sp.) includes the thermophilous scrub vegetation of the *Scutellario-Urticetum rupestris* (*Pruno spinosae-Rubion ulmifolii* alliance, *Crataego-Prunetea* class). In this last association, *U. rupestris*, together with *Scutellaria rubicunda* Hornem., was indicated by Brullo et al. [34] as a characteristic species for the Hyblaean territory [35]. It is a nemoral and sciaphilous association characterized by species belonging to the *Rhamno-Prunetea* class, such as *Rubus ulmifolius* Schott, *Smilax aspera* L., *Clematis cirrhosa* L., *C. vitalba* L., and *Crataegus monogyna* Jacq. This phytocoenosis is essentially localized in the thermo-mesophilious woods of the *Doronico-Quercetum ilicis*, *Ostryo-Quercetum ilicis*, and *Pistacio-Quercetum ilicis*. Most species of this phytocoenosis belong to the Mediterranean element (45%), with the dominant life form corresponding to phanerophytes/nanophanerophytes (34%) and hemicryptophytes (31%).

Cluster A includes perennial vegetation growing mainly on dripping vertical limestone cliffs and shady ravines, which are humid for most of the year, in the shade of wooded formations that are dominated by *Quercus ilex* within the Hyblaean caves. The structure of the community is determined by *U. rupestris*, together with several hygrophilous species of bryophytes and pteridophytes, such as *Pellia endiviifolia*, *Thamnobryum alopecurum* (Hedw.) Gangulee, *Asplenium scolopendrium* subsp. *scolopendrium*, *Dryopteris filix-mas* (L.) Schott, and *Adiantum capillus-veneris*, *Asplenium sagittatum* (DC.) Bange. Moreover, this vegetation is enriched with several lianose species, such as *Hedera helix* L., *Rubia peregrina* L. *Dioscorea communis* (L.) Caddick & Wilkin, *Clematis vitalba*, and *Aristolochia sempervirens* L. Due to its ecological features, *U. rupestris* has been proposed as a characteristic species of a new association named *Urtico rupestris-Adiantetum capilli-veneris* ass. nova hoc loco (* holotypus: Table 1, Rel. 10) within the *Polysticho setiferi-Phyllitidion scolopendrii* alliance (*Adiantetea capilli-veneris* class, Appendix B). From a chorological and structural viewpoint, this vegetation highlights the relevance of this species with a Mediterranean distribution (29%), with hemicryptophytes (53%) being the dominant life forms. This new association shows floristic-ecological affinities with *Thamnobryo alopecuri-Phyllitidetum scolopendrium* Brullo, Privitera & Puglisi 1993, which have been described for southern Italy and Sicily [27]. Furthermore, it shows edaphic-ecological characteristics similar to *Conocephalo conici-Woodwardietum radicantis* Brullo, Lo Giudice, & Privitera 1989, *Adianto capilli veneris-Pteridetum vittatae* Brullo, Lo Giudice & Privitera 1989, *Adianto capilli veneris-Osmundetum regalis* Brullo, Lo Giudice & Privitera 1989. These chasmo-comophytic associations are rich in bryophytes and pteridophytes of shady/humid cliffs, which were described for the Peloritani Mountains in the north-eastern sector of Sicily [26]. From a bioclimatic point view, the *Urtico rupestris-Adiantetum capilli-veneris* falls within the upper Thermomediterranean and lower Mesomediterranean belts with lower dry and upper ombrotypes [36].

### 2.4. Distribution and Conservation Status

**Figure 4:**
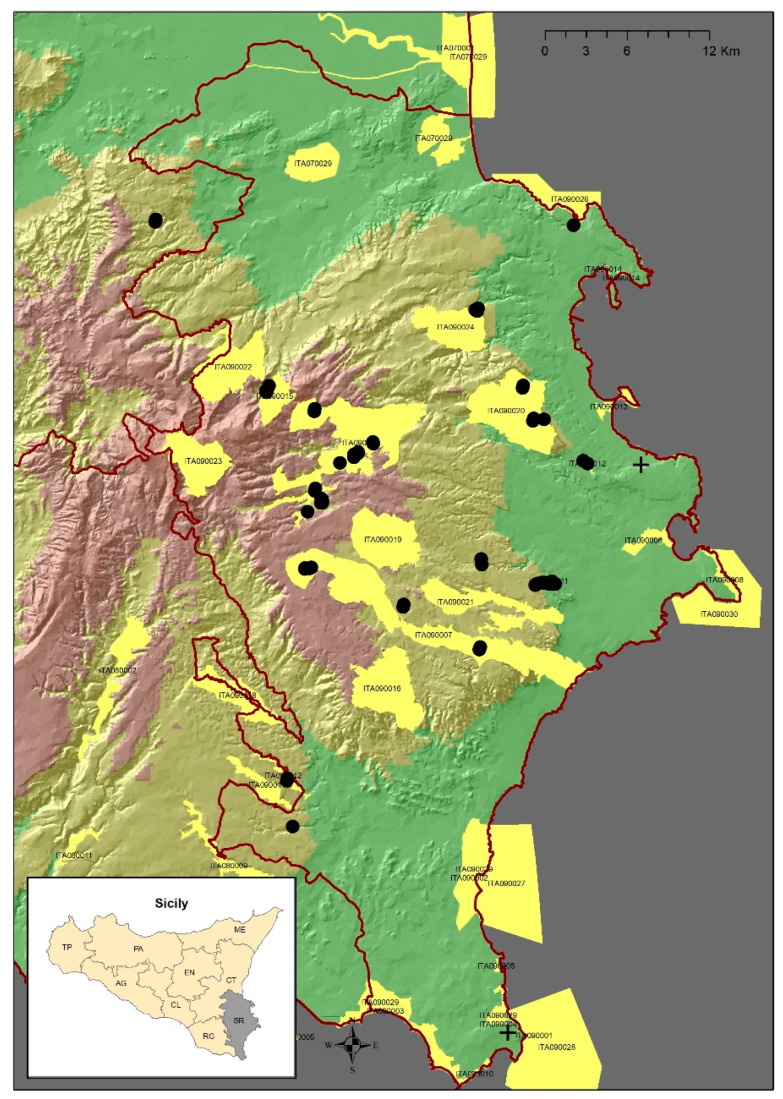
Geographical distribution of *Urtica rupestris* in Sicily. Black dot, current distribution; black cross, no longer recorded; yellow polygons, Special Areas Conservation.

Our investigations confirmed 13 sites with *U. rupestris* (Figure 4): 1. Vallone Carcarone—Militello (1 plot); 2. Torrente Belluzza—Villasmundo (1 plot, SAC-ITA090024); 3. Cava Sorciaro, Cava Mostringiano—Monti Climiti (3 plots, SAC-ITA090020); 4. Grotta Palombara—Siracusa (1 plot, SAC-ITA090012); 5. Grotta Monello—Siracusa (1 plot, SAC-ITA090011; 1 plot out of the SAC); 6. Vallone Caradonna—Canicattini Bagni (1 plot); 7. Cava Grande del Cassibile e Cava di Baulì, Manghisi (2 plots, SAC-ITA090007; 2 plots out of the SAC); 9. Cava del Prainito, Rosolini (1 plot, SAC-ITA080012); 10. Cava Grande, Valle dell’Anapo e Bibinello—Buscemi, Cassaro, Ferla, Palazzolo Acreide, Sortino (5 plots, SAC-ITA090009); 11. Sant’Andrea, Valle Cupa—Buccheri (1 plot, SAC-ITA090015); 12. Cava Rosolini (La Rosa A., Aprile. 2016) (1 plot); 13. Cava Brucoli (Alicata I., 6.11.2016) (1 plot). The species was no longer found in two of the sites: first in Syracuse “*scendendo dal Belvedere a oriente verso il mare*” (Lojacono 1904) and second in “*Pantani Capo Passero*” (Lopriore 1900). In total, *U. rupestris* falls within 22 cells (2 × 2 km) and eight special areas of conservation (SAC), and outside of four Natura 2000 sites. The area hosting the largest number of individuals was the N2000 “Valle del Fiume Anapo, Cavagrande del Calcinara, Cugni di Sortino” (ITA090009) site. Currently, almost all *U. rupestris* sites are located in the Syracuse province, with the exception of the *locus classicus*, which falls within the Catania territory, and one in the Ragusa province. Despite the many records of the distribution of the species, *U. rupestris* is threatened by many factors that, over time, have altered and reduced its natural habitat. *Urtica rupestris* was listed in the Red Book of Italian plants [37] as lower risk (LR), which was subsequently reconsidered by Brullo et al. [13] as a vulnerable species (VU), and, more recently, has been classified as a vulnerable species [19,20]. Our accurate field surveys allowed us to have a deeper knowledge of the distribution and conservation status of *U. rupestris*. Considering our current assessments and observations in the field, the species is currently recorded in 22 cells in the Hyblaean territory. According to the reference grid for Italy [38] and the GeoCAT tool, based on the IUCN criterion B, we propose a new status for this species, which should be considered endangered (EN) B2ab (ii, iii). In fact, we assessed this using the GeoCAT web tool, and calculated an EOO area equal to 1880 km^2^ and an AOO equal to 104 km^2^. This decline in the original population, because of habitat loss and fragmentation, suggests that this species could be classified as endangered (EN).

## 3. Materials and Methods

Data in the literature regarding the distribution of this species were reviewed [39,40,41,42,43], and specimens of herbaria of Catania (CAT) and Palermo (PA) were also examined. Based on these starting data, all the sites in which *U. rupestris* has been reported were visited. In addition, other sites that were potentially suitable for the species were investigated. In order to analyze the floristic composition of the *U. rupestris* plant community in the Hyblaean territory, several field activities were carried out in the years between 2015 and 2020. A total of 52 relevés were collected, of which 34 were unpublished and 18 from bibliographic data [34,44,45]. The floristic composition and cover of the species in each plot were determined using the standard phytosociological method [46]. All the phytosociological relevés were processed using classification and ordination methods. The numerical analysis was performed using the software package “PCORD” 6.08 and R software. A multivariate analysis (linkage method: Ward’s, distance measure: Bray–Curtis) was applied. Clustering was performed using the R package “pvclust” [47]. Pvclust computes *p*-values for each cluster’s uncertainty using bootstrap resampling. The bootstrap sample size was set to 1000. Detrended correspondence analyses (DCA) were utilized in order to develop a hypothesis about the vegetation/environmental interactions and to establish geographic patterns in the scatter-gram [48]. The DCA takes into account different quantitative data, such as the vegetation coverage (%),number of species (N. sp.), altitude (m a.s.l.), and slope (◦). Quantum GIS software version 3.6 and GPS Garmin Montana were used to geolocalize the surveyed population. For the risk assessment at the regional scale (Sicily), we followed the IUCN protocol and the most recent guidelines for its application [49]. In particular, we applied the IUCN criterion B for estimating the trends in the Area of Occupancy (AOO) using the 2 × 2 km grid for the Italian territory proposed by Gargano [38]. On the other hand, in order to obtain an accurate assessment, we also calculated the Area of Occupancy (AOO, km^2^) and Extent of Occurrence (EOO) using the Geo-CAT web tool (Geospatial Conservation Assessment Tool) programme [50], which performs a rapid geospatial analysis of species in a simple way.

The identification of vascular plants was carried out according to Pignatti et al. [51,52,53,54], and the nomenclature follows the Portal to the Flora of Italy (http://dryades.units.it/floritaly/ accessed on 15 October 2022) [55], whereas the nomenclature of bryophytes is in accordance with Cortini Pedrotti [56,57]. The syntaxonomical nomenclature follows Mucina et al. [58]. The bioclimatic units refer to Bazan et al. [36].

### Study Area

The study area, located in the southeast of Sicily, is represented by the Hyblaean plateau which belongs to the African plate. It comprises of a crust of continental types different from that of the rest of Sicily [59], whereas, from a geophysicist standpoint, it is characterized by a strong gravimetric and magnetic anomaly (Bouguer anomalies) chiefly due to its composition. Outcropping successions in the Hyblaean plateau consist mostly of carbonate and carbonate-marly sediments ranging from Lower Cretaceous to Pleistocene, where basic volcanics of considerable power [60] are intercalated. One of the most typical landscapes of the Hyblaean area is the “Cave”, which are valleys of fluvial-tectonic origin, with a cross-section very similar to a V with steep slopes. Water courses flowing in the “Cave” usually have temporary arrangements or a permanent regime. Furthermore, the Hyblean territory is characterized by the presence of very important coastal wetlands [61,62,63,64], as well as several rocky pools and temporary ponds that host very specialized vascular flora [65,66,67,68,69]. Blasi et al. [70] identified six important plant areas (IPAs) for the Hyblaean territory, which are essential for the conservation of plant biodiversity. Furthermore, the Hyblaean territory is affected by 43 special areas of conservation (SAC), and 9 regional protected areas. According to the bioclimatic classification proposed by Rivas-Martínez [71,72], the area under study is referred to as the Mediterranean pluviseasonal oceanic bioclimate, with thermotypes ranging from low thermomediterranean to supramediterranean, and ombrotypes from semiarid to lower humid.

## 4. Conclusions

The ecological and phytosociological analyses carried out on *U. rupestris* in the Sicilian territory pointed out the biogeographical importance of this rupicolous species and of the shady dripping cliff habitat that deserves to be included in Annex I of the 93/42 EEC Directive. The re-assessment of the conservation status of this species (EN) highlights the urgent need to primarily preserve the habitats of the “Hyblaean Cave”, which hosts *U. rupestris* and several other restricted endemic species, such as *Zelkova sicula*, *Trachelium caeruleum* subsp. *lanceolatum*, *Anthemis pignattiorum*, *Limonium pachynense*, *L. pavonianum*, *Epipactis hyblaea*, etc. Therefore, the outcomes of this research can be included in future conservation and management strategies for this rare endemic taxon. 

## Figures and Tables

**Table 1:** *Urtico rupestris-Adiantetum capilli-veneris* ass. nova hoc loco. Phytosociological relevés of the plant community investigated. The symbol * display the holotypus.

		Cluster number	1	2	3	4	5	14	15	16	17	18	19	20	21	22	23	24	35	36	49	50	
		Relevé number	1	2	3	4	5	6	7	8	9	10 *	11	12	13	14	15	16	17	18	19	20	
		Area (mq)	16	16	16	16	16	16	16	16	16	16	16	16	16	16	16	16	3	6	3	6	
		Coverage (%)	85	80	85	95	35	90	90	85	85	95	95	90	90	90	90	80	100	100	80	100	
		Aspect	N	N	N	N	N	N	N	-	NE	NE	-	NE	-	-	-	-	-	-	-	-	Presence
		Slope (◦)	45	35	30	35	85	90	80	80	75	90	90	20	70	80	80	70	80	60	80	100	
		Altitude	341	345	351	355	140	461	461	460	465	465	465	465	465	465	465	464	320	320	320	320	
Chorotype	Life form	Floristic richness	20	14	18	16	10	20	20	22	16	19	15	16	22	22	20	21	10	12	10	11	
		**Char. Ass**																					
End. Sic.	H	*Urtica rupestris* Guss.	3	2	2	4	1	3	4	2	3	4	3	3	3	1	2	3	3	3	5	3	20
		**Char. *Polysticho setiferi-Phyllitidion scolopendrii* & *Adiantetea capilli-veneris***																					
Circumbor.	H	*Asplenium scolopendrium* L. subsp. *scolopendrium*	2	2	1	1	.	+	+	2	3	2	3	3	2	3	3	3	.	2	1	2	18
Subtrop.	G	*Adiantum capillus-veneris* L.	1	2	1	+	+	2	+	2	1	1	1	2	2	1	+	1	+	.	.	.	17
Southern-temp.		*Pellia endiviifolia* (Dicks.) Dum.	1	+	1	+	.	+	1	+	+	1	1	+	1	+	1	1	.	.	.	.	15
Temperate		*Thamnobryum alopecurum* (Hedw.) Gangulee	1	1	1	1	.	1	2	1	1	+	1	1	2	1	1	1	.	.	.	.	15
Steno-Medit.-Merid.	H	*Asplenium sagittatum* (DC.) Bange	.	.	.	.	.	.	.	.	.	.	.	.	.	.	.	.	.	.	+	.	1
		**Other species**																					
NW-Medit.	H	*Lamium flexuosum* Ten.	1	2	3	2	.	+	1	2	+	+	2	1	1	2	3	1	1	2	.	2	18
Steno-Medit.-Occ.	H	*Acanthus mollis* L.	+	+	+	+	+	1	2	1	+	+	.	.	+	+	1	+	1	2	+	2	18
Subatl.	P	*Hedera helix* L.	+	1	1	+	1	2	1	1	+	2	1	+	.	.	.	.	4	3	2	3	16
Euri-Medit.	G	*Dioscorea communis* (L.) Caddick & Wilkin	1	.	1	+	1	.	.	.	.	.	.	1	+	+	+	+	+	2	2	2	13
Steno-Medit.	P	*Rubia peregrina* L.	+	.	.	.	.	+	1	+	+	1	+	+	+	+	+	+	.	.	.	.	12
Paleotemp.	H	*Brachypodium sylvaticum* (Huds.) P. Beauv.	1	+	1	+	.	+	+	+	.	+	.	.	+	+	1	+	.	.	.	.	12
Temperate		*Plagiomnium affine* (Blandow ex Funck) T.J.Kop.	.	.	.	.	.	+	+	+	+	1	1	+	+	+	1	1	.	.	.	.	11
Eurasiat.	H	*Ficaria verna* Huds. subsp. *verna*	.	.	.	.	.	+	1	+	+	+	1	+	1	+	+	+	.	.	.	.	11
Euri-Medit.	NP	*Rubus ulmifolius* Schott	.	.	1	1	.	+	1	1	.	.	.	.	+	1	+	+	.	.	2	.	10
Subcosmop.	T	*Geranium robertianum* L.	1	1	1	1	.	.	.	.	.	.	.	.	+	+	+	+	+	1	.	.	10
Endem. Sic.	Ch	*Cymbalaria pubescens* (C. Presl) Cufod.	2	.	.	+	1	1	2	+	+	1	.	.	.	.	.	.	.	+	1	.	10
Medit-Atl.		*Scorpiurium circinatum* (Brid.) M. Fleisch. & Loeske	.	.	.	.	.	1	1	1	+	+	+	+	.	1	.	+	.	.	.	.	9
Steno-Medit.	NP	*Hypericum hircinum* L.	+	.	+	.	.	+	.	1	.	.	+	+	1	2	1	.	.	.	.	.	9
Europ.-Caucas.	P	*Sambucus nigra* L.	.	.	.	.	.	.	.	1	+	+	+	1	+	+	+	.	.	.	.	.	8
Steno-Medit.	G	*Arum italicum* Mill.	.	.	.	.	.	.	+	+	+	1	+	+	.	.	.	+	.	.	.	.	7
Steno-Medit.	T	*Parietaria lusitanica* L.	1	1	1	1	.	.	.	.	.	.	.	.	.	.	.	.	1	+	.	.	6
Steno-Medit.	G	*Umbilicus horizontalis* (Guss.) DC.	.	.	.	.	.	1	1	+	.	+	+	+	.	.	.	.	.	.	.	.	6
Europ.	P	*Clematis vitalba* L.	.	.	.	.	1	+	+	+	.	+	.	.	.	.	.	+	.	.	.	.	6
Subcosmop	G	*Dryopteris filix-mas* (L.) Schot	+	.	.	+	.	1	1	.	.	.	.	.	+	.	.	.	.	.	.	.	5
Endem. Ital.	H	*Scutellaria rubicunda* Hornem.	.	.	.	.	.	.	.	.	.	.	.	.	+	+	.	.	+	+	.	+	5
Pontica	H	*Anthriscus nemorosa* (M. Bieb.) Spreng.	+	+	+	+	.	.	.	.	.	.	.	.	.	.	.	1	.	.	.	.	5
Steno-Medit.	H	*Melissa officinalis* subsp. *altissima* (Sm.) Arcang.	1	.	1	1	.	+	1	.	.	.	.	.	.	.	.	.	.	.	.	.	5
Euri-Medit.	Ch	*Ruscus aculeatus* L.	.	.	.	.	.	.	.	.	+	+	.	.	.	+	+	.	.	.	.	.	4
Steno-Medit.	H	*Viola alba* subsp. *dehnhardtii* (Ten.) W. Becker	.	.	.	.	.	.	.	.	.	.	.	.	+	+	+	+	.	.	.	.	4
Steno-Medit.	H	*Carex distachya* Desf.	.	.	.	.	.	.	.	+	.	.	.	.	+	+	+	.	.	.	.	.	4
Eurasiat.	H	*Carex pendula* Huds.	+	+	1	.	.	.	.	.	.	.	.	.	.	.	.	.	.	.	.	.	3
Paleotemp.	H	*Eupatorium cannabinum* L. subsp. *cannabinum*	.	.	.	.	.	.	.	.	.	.	.	.	+	+	.	+	.	.	.	.	3
Eurasiat.	T	*Galium aparine* L.	.	.	.	.	.	.	.	.	.	.	.	.	.	.	.	.	+	1	.	+	3
Medit-Atl.		*Lunularia cruciata* (L.) Dumort. ex Lindb.	1	+	1	.	.	.	.	.	.	.	.	.	.	.	.	.	.	.	.	.	3
Euri-Medit.	T	*Geranium purpureum* Vill.	.	.	.	.	.	.	.	.	.	.	.	.	.	.	.	.	.	.	1	+	2
Subtrop.	H	*Asplenium onopteris* L.	.	.	.	.	+	.	.	+	.	.	.	.	.	.	.	.	.	.	.	.	2
Circumbor.	H	*Chelidonium majus* L.	.	.	.	.	.	.	.	.	.	.	.	.	.	.	.	.	.	+	.	+	2
Steno-Medit.-Occ.	G	*Allium subhirsutum* L.	.	.	.	.	+	.	.	.	.	.	.	.	.	.	.	.	.	.	.	.	1
Medit.-Turan.	P	*Ficus carica* L.	.	.	.	.	+	.	.	.	.	.	.	.	.	.	.	.	.	.	.	.	1
Euri-Medit.	H	*Parietaria judaica* L.	.	.	.	.	.	.	.	.	.	.	.	.	.	.	.	.	.	.	.	+	1
Euri-Medit.	H	*Polypodium cambricum* L.	.	.	.	.	.	.	.	.	.	.	.	.	.	.	.	+	.	.	.	.	1
Circumbor.	G	*Polystichum setiferum* (Forssk.) T. Moore ex Woyn.	.	.	.	.	.	.	.	.	.	.	.	.	.	.	.	.	.	.	+	.	1
Subcosmop.	H	*Potentilla reptans* L.	.	.	.	.	.	.	.	.	.	.	.	.	+	.	.	.	.	.	.	.	1
Subcosmop.	H	*Urtica dioica* L.	.	.	.	.	.	.	.	.	.	.	.	.	.	.	1	.	.	.	.	.	1

## Data Availability

Not applicable.

## Supplementary Materials

The following are available online at https://www.mdpi.com/article/10.3390/plants12010164/s1, Table S1: Phytosociological relevés; Scheme S1: Old records and Specimina visa of *U. rupestris*.

## Appendix A. Localities and Dates of Relevés (Table 1)

Rel. 1, Cava Grande del Cassibile, 6.05.2014 (Minissale & Sciandrello); Rel. 2–4, Cava Grande del Cassibile, 09.06.2014 (Minissale & Sciandrello); Rel. 5, 07.04.2015, Villasmundo, Belluzza (Sciandrello); Rel. 6–15, Cava Bibinello, Palazzolo Acreide, 30.03.2021 (Cambria, Minissale, Sciandrello); Rel. 16–17, Pantalica (Minissale et al. 2007); Rel. 18–19, Pantalica (Brullo et al. 1993).

## Appendix B. Syntaxonomical Scheme

*Adiantetea capilli-veneris* Br.-Bl. in Br.-Bl., Roussine & Negre 1952
*Adiantetalia capilli-veneris* Br.-Bl. ex Horvatic 1934
*Polysticho setiferi-Phyllitidion scolopendri* Ubaldi ex Ubaldi & Biondi in Biondi, Allegrezza, Casavecchia, Galdenzi, Gasparri, Pesaresi, Vagge & Blasi 2014
*Urtico rupestris-Adiantetum capilli-veneris ass. nova hoc loco*
*Crataego-Prunetea spinosae* R.Tx. 1962
*Pyro spinosae-Rubetalia ulmifolii* Biondi, Blasi & Casavecchia in Biondi et al. 2014
*Pruno spinosae-Rubion ulmifolii* O. Bolòs 1954
*Scutellario rubicundae-Urticetum rupestris* Brullo, Minissale, Scelsi & Spamp. 1993.

## References

[1] Schröter D., Cramer W., Leemans R., Prentice I.C., Araújo M.B., Arnell N.W., Bondeau A., Bugmann H., Carter T.R., Gracia C.A. (2005). Ecosystem service supply and vulnerability to global change in Europe. Science.

[2] Tomaselli V., Tenerelli P., Sciandrello S. (2012). Mapping and quantifying habitat fragmentation in small coastal areas: A case study of three protected wetlands in Apulia (Italy). Environ. Monitor. Assessm..

[3] Médail F., Quézel P. (1997). Hot-spots analysis for conservation of plant biodiversity in the Mediterranean Basin. Ann. Mo. Bot. Gard..

[4] Médail F., Quézel P. (1999). Biodiversity Hotspots in the Mediterranean Basin: Setting global conservation priorities. Conserv. Biol..

[5] Sciandrello S., Guarino R., Minissale P., Spampinato G. (2015). The endemic vascular flora of Peloritani Mountains (NE Sicily): Plant functional traits and phytogeographical relationships in the most isolated and fragmentary micro-plate of the Alpine orogeny. Plant Biosyst..

[6] Sciandrello S., Cambria S., Giusso del Galdo G., Guarino R., Minissale P., Pasta S., Tavilla G., Cristaudo A. (2021). Floristic and Vegetation Changes on a Small Mediterranean Island over the Last Century. Plants.

[7] Sciandrello S., Minissale P., Giusso del Galdo G. (2020). Vascular plant species diversity of Mt. Etna (Sicily): Endemicity, insularity and spatial patterns along the altitudinal gradient of the highest active volcano in Europe. PeerJ.

[8] Brullo S., Cambria S., Crisafulli A., Tavilla G., Sciandrello S. (2021). Taxonomic remarks on the *Centaurea aeolica* (Asteraceae) species complex. Phytotaxa.

[9] Tavilla G., Angiolini C., Bagella S., Bonini F., Cambria S., Caria M.C., Esposito A., Fanfarillo E., Ferri V., Fiaschi T. (2022). New national and regional Annex I Habitat records: From #37 to #44. Plant Sociol..

[10] Bartolucci F., Peruzzi L., Galasso G., Albano A., Alessandrini A., Ardenghi N.M.G., Astuti G., Bacchetta G., Ballelli S., Banfi E. (2018). An updated checklist of the vascular flora native to Italy. Plant Biosyst..

[11] Peruzzi L., Conti F., Bartolucci F. (2014). An inventory of vascular plants endemic to Italy. Phytotaxa.

[12] Brullo S., Grillo M., Guglielmo A. (1998). Considerazioni fitogeografiche sulla flora iblea. Boll. Acc. Sci. Nat..

[13] Brullo C., Minissale P., Sciandrello S., Spampinato G. (2011). Phytogeographic survey on the endemic vascular flora of the Hyblaean territory (SE Sicily-Italy). Acta Bot. Gall..

[14] Pavone P., Spampinato G., Tomaselli V., Sciandrello S., Ronsisvalle F. (2007). Map of the habitats of the EEC Directive 92/43 in the biotopes of the Syracuse province (eastern Sicily). Fitosociologia.

[15] Garfì G., Carimi F., Pasta S., Rühl J., Trigila S. (2011). Additional insights on the ecology of the relic tree *Zelkova sicula* Di Pasquale, Garfì & Quézel (Ulmaceae) after the finding of a new population. Flora.

[16] Guarino R., Raimondo F.M., Domina G. (2013). A new species of Anthemis sect. Hiorthia (Asteraceae) from SE Sicily. Plant Biosyst..

[17] Brullo S., Brullo C., Sciandrello S., Tavilla G., Cambria S., Tomaselli V., Ilardi V., Giusso Del Galdo G., Minissale P. (2022). The Plant Communities of the Class *Isoëto-Nanojuncetea* in Sicily. Plants.

[18] Grosse-Veldmann B., Nürk N.M., Smissen R., Breitwieser I., Quandt D., Weigend M. (2016). Pulling the sting out of nettle systematics—A comprehensive phylogeny of the genus *Urtica* L. (Urticaceae). Mol. Phylogenet. Evol..

[19] Rossi G., Orsenigo S., Gargano D., Montagnani C., Peruzzi L., Fenu G., Abeli T., Alessandrini A., Astuti G., Bacchetta G. (2020). Lista Rossa Della Flora Italiana. 2 Endemiti e Altre Specie Minacciate.

[20] Orsenigo S., Fenu G., Gargano D., Montagnani C., Abeli T., Alessandrini A., Bacchetta G., Bartolucci F., Carta A., Castello M. (2021). Red list of threatened vascular plants in Italy. Plant Biosyst..

[21] Corsi G., Garbari F., Maffei F. (1999). Il genere *Urtica* L. (Urticaceae) in Italia. Revisione biosistematica. Webbia.

[22] Brullo S., Costanzo E., Tomaselli V. (2001). Étude phytosociologique sur les formations à Laurus nobilis L. dans le Monts Iblei (Sicile sud-orientale). Phytocoenologia.

[23] Foucault B. (2015). Contribution au prodrome des végétations de France: Les *Adiantetea capilli-veneris* Braun-Blanq. ex Braun-Blanq., Roussine & Nègre 1952. Acta Bot. Gall..

[24] Biondi E., Blasi C., Allegrezza M., Anzellotti I., Azzella M.M., Carli E., Casavecchia S., Copiz R., Del Vico E., Facioni L. (2014). Plant communities of Italy: The Vegetation Prodrome. Plant Biosyst..

[25] Biondi E., Allegrezza M., Casavecchia S., Galdenzi D., Gasparri R., Pesaresi S., Vagge I., Blasi C. (2014). New and validated syntaxa for the checklist of Italian vegetation. Plant Biosyst..

[26] Brullo S., Lo Giudice R., Privitera M. (1989). La classe *Adiantetea* in Sicilia. Arch. Bot. Ital..

[27] Brullo S., Privitera P., Puglisi M. (1993). *Thamnobryo alopecuri-Phyllitidetum scolopendrium* nuova associazione centro-mediterranea della classe *Adiantetea*. Arch. Bot. Ital..

[28] Brullo S., Scelsi F., Spampinato G. (2001). La Vegetazione dell’Aspromonte. Studio Fitosociologico.

[29] Cortini Pedrotti C. (1982). Associations de la classe Adiantetea dans quelques grottes de la gorge de Frasassi. Guide-Itinéraire. Excursion Internationale de Phytosociologie en Italie centrale (2-11 juillet 1982). Univ. Di Camerino.

[30] Puglisi M. (1994). *Homalio lusitanicae-Adiantetum*, nuova associazione della classe *Adiantetea* Br.-Bl. 1947. Boll. Acc. Gioenia Sci. Nat..

[31] Spampinato G., Genovesi P., Angelini P., Bianchi E., Dupré E., Ercole S., Giacanelli V., Ronchi F., Stoch F. (2014). Rupi Stillicidiose Mediterranee dell’Adiantion: Nuovo habitat proposto per l’inserimento nell’allegato i della Direttiva (92/43CEE). Specie e Habitat di Interesse Comunitario in Italia: Distribuzione, Stato di Conservazione e Trend.

[32] Guarino R., Pasta S., Bazan G., Crisafulli A., Caldarella O., Giusso del Galdo G., Silvestre Gristina A., Ilardi V., La Mantia A., Marcenò C. (2021). Relevant habitats neglected by the Directive 92/43 EEC: The contribution of Vegetation Science for their reappraisal in Sicily. Plant Sociol..

[33] Sciandrello S., Cambria S., Crisafulli A., Giusso del Galdo G., Minissale P., Musarella C.M., Puglisi M., Tavilla G., Tomaselli V., Spampinato G. A new habitat of the shady wet cliffs (Adiantetea capilli-veneris) of the Mediterranean region. Proceedings of the 54th SISV Congress, Twenty years in the third millennium with Vegetation Science.

[34] Brullo S., Minissale P., Scelsi F., Spampinato G. (1993). Note fitosociologiche miscellanee sul territorio ibleo (Sicilia sud-orientale). Boll. Accad. Gienia Sci. Nat..

[35] Bartolo G., Brullo S., Fichera G., Scelsi F. (1989). Osservazioni fitosociologiche sulla vegetazione a *Urtica rupestris* Guss. del territorio ibleo (Sicilia sudorientale). Giorn. Bot. Ital..

[36] Bazan G., Marino P., Guarino R., Domina G., Schicchi R. (2015). Bioclimatology and vegetation series in Sicily: A geostatistical approach. Ann. Bot. Fenn..

[37] Conti F., Manzi A., Pedrotti F. (1997). Liste rosse Regionali delle Piante d’Italia.

[38] Gargano D. (2011). Proposta metodologica. Verso la realizzazione di nuove Liste Rosse della flora d’Italia: Una griglia standard per la misura dell’Area of Occupancy (AOO). Inf. Bot. Ital..

[39] Giardina G., Raimondo F.M., Spadaro V. (2007). A catalogue of plants growing in Sicily. Bocconea.

[40] Gussone J. (1821). Catalogus Plantarum Quae Observantur in Regio Horto ser. Fr. Borbonii Principis Juventutis in Boccadifalco prope Panormum.

[41] Gussone J. (1845). Florae Siculae Synopsis 2 (2).

[42] Lopriore C. (1900). Studi Comparativi Sulla Flora Lacustre Della Sicilia.

[43] Lojacono Pojero M. (1904). Flora Sicula o Descrizione Delle Piante Vascolari Spontanee o Indigenate in Sicilia.

[44] Minissale P., Sciandrello S., Spampinato G. (2007). Analisi della biodiversità vegetale e relativa cartografia della Riserva Naturale Orientata “Pantalica, Valle dell’Anapo e Torrente Cava Grande” (Sicilia sudorientale). Quad. Bot. Amb. Appl..

[45] Zimmitti A., Ronsisvalle F.B.F., Ronsisvalle G.A. (2007). Aree di interesse naturalistico per la rete ecologica dei M.ti Iblei (Sicilia sud-orientale): Il territorio dei Monti Climiti. Quad. Bot. Amb. Appl..

[46] Braun-Blanquet J. (1964). Pflanzensoziologie. Grundzüge der Vegetationskunde.

[47] Suzuki R., Shimodaira H. (2006). Pvclust: An R package for assessing the uncertainty in hierarchical clustering. Bioinformatics.

[48] Hill M.O., Gauch H.G. (1980). Detrended correspondence analysis: An improved ordination technique. Vegetatio.

[49] IUCN Standards and Petitions Committee Guidelines for Using the IUCN Red List Categories and Criteria. Version 15.1. Prepared by the Standards and Petitions Committee. https://www.iucnredlist.org/documents/RedListGuidelines.pdf.

[50] Bachman S., Moat J., Hill A.W., Torre J., Scott B. (2011). Supporting Red List threat assessments with GeoCAT: Geospatial conservation assessment tool. ZooKeys.

[51] Pignatti S., Guarino R., La Rosa M. (2017). Volume 1: Flora d’Italia & Flora Digitale. In Flora d’Italia: In 4 Volumi.

[52] Pignatti S., Guarino R., La Rosa M. (2017). Volume 2: Flora d’Italia & Flora Digitale. In Flora d’Italia: In 4 Volumi.

[53] Pignatti S., Guarino R., La Rosa M. (2018). Volume 3: Flora d’Italia & Flora Digitale. In Flora d’Italia: In 4 Volumi.

[54] Pignatti S., Guarino R., La Rosa M. (2019). Volume 4: Flora d’Italia & Flora Digitale. In Flora d’Italia: In 4 Volumi.

[55] Martellos S., Bartolucci F., Conti F., Galasso G., Moro A., Pennesi R., Peruzzi L., Pittao E., Nimis P.L. (2020). FlorItaly—The portal to the Flora of Italy. PhytoKeys.

[56] Cortini Pedrotti C. (2001). Flora dei Muschi d’Italia. Sphagnopsida, Andreaeopsida, Bryopsida (I Parte).

[57] Cortini Pedrotti C. (2005). Flora dei Muschi d’Italia. Bryopsida (II Parte).

[58] Mucina L., Bültmann H., Dierßen K., Theurillat J.-P., Raus T., Carni A., Šumberová K., Willner W., Dengler J., García R.G. (2016). Vegetation of Europe: Hierarchical floristic classification system of vascular plant, bryophyte, lichen, and algal communities. Appl. Veg. Sci..

[59] Ben Avraham Z., Grasso M. (1990). Collisional zone segmentation in Sicily and surrounding areas in the Central Mediterranean. Ann. Tecton..

[60] Bianchi F., Carbone S., Grasso M., Invernizzi G., Lentini F., Longaretti G., Merlini G., Mostardini F. (1989). Sicilia orientale: Profilo geologico Nebrodi-Iblei. Mem. Soc. Geol. Ital..

[61] Minissale P., Santo A., Sciandrello S. (2011). The coastal vegetation of the SCI “Capo Murro di Porco, Penisola della Maddalena e Grotta Pellegrino” (Siracusa). Fitosociologia.

[62] Sciandrello S., Guglielmo A., Spampinato G. (2014). Spatial patterns and floristic composition of plant communities in coastal salt marshes of south-eastern Sicily (Italy). Acta Bot. Gall..

[63] Sciandrello S., Musarella C.M., Puglisi M., Spampinato G., Tomaselli V., Minissale P. (2019). Updated and new insights on the coastal halophilous vegetation of southeastern Sicily (Italy). Plant Sociol..

[64] Sciandrello S. (2020). Coastal saltmarsh vegetation in Sicily (Italy): Phytosociological insights and plant diversity. Plant Biosyst..

[65] Minissale P., Molina J.A., Sciandrello S. (2017). *Pilularia minuta* Durieu (Marsileaceae) discovered in south-eastern-Sicily: New insights on its ecology, distribution and conservation status. Bot. Lett..

[66] Minissale P., Sciandrello S. (2016). Ecological features affect patterns of plant communities in Mediterranean temporary rock pools. Plant Biosyst..

[67] Minissale P., Trigilia A., Brogna F., Sciandrello S. (2015). Plants and vegetation in the archaeological park of Neapolis of Siracusa (Sicily–Italy). A management effort but also an opportunity for a better enjoyment of the site. Conserv. Manag. Archeol..

[68] Sciandrello S., Privitera M., Puglisi M., Minissale P. (2016). Plant communities diversity and spatial patterns in volcanic temporary pond of Sicily (S-Italy). Biologia.

[69] Brullo S., Brullo C., Tavilla G., Cambria S., Minissale P., Sciandrello S., Giusso del Galdo G., Siracusa G., Del Guacchio E. (2022). About the occurrence of *Elatine macropoda* and *E. gussonei* (Elatinaceae) in Sicily and lectotypification of their names. Acta Bot. Croat..

[70] Blasi C., Marignani M., Copiz R., Fipaldini M., Bonacquisti S., Del Vico E., Rosati L., Zavattero L. (2011). Important plant areas in Italy: From data to mapping. Biol. Conserv..

[71] Rivas Martínez S. (1993). Bases para una nueva clasificacion bioclimatica de la tierra. Folia Bot. Matritensis.

[72] Rivas-Martínez S. (2004). Bioclimatic Map of Europe: Bioclimates, Scale 1:16 Mill.

