# Supplementary material for: Ecological Features and Conservation of Urtica rupestris Guss. (Urticaceae): A Narrow Endemic Species of Sicily
Source: Plants (Basel). 2022 Dec 29;12(1):164. doi: 10.3390/plants12010164 (PMC9824435; doi:10.3390/plants12010164)
Supplement: Supplementary file 1 [file plants-12-00164-s001.zip › Scheme S1_Old record and specimina visa_Urtica.pdf]

Old records of *U. rupestris*

Sortino nel Vallone della Preola, ad Avola (SR) (Gussone, 1821); Avola Cava dell'Amico (Bianca) (Gussone 1845); Pantani Capo Passero (Lopriore 1900); Luoghi cavernosi ombrosi sulle rupi calcaree ombreggiate, rara e locale del mezzogiorno dell'Isola, Militello di V. di Noto nel Vallone del Calcarone Guss. Tin.; Siracusa scendendo dal Belvedere ad oriente verso il mare, nei terreni rupestri cavernosi Lojac.; Sortino Vallone della Preola Tin. Avola Bianca, Aprile-Maggio (Lojacono 1904); Belvedere presso Siracusa (Lojacono-Pojero, 1907); Cava Monstringiano sui Climiti, Cava Grande del Calcinara, Valle dell'Anapo (Brullo et al. 1993); Militello Val di Noto nel V.ne del Carcarone (Gasparrini PAV, Gussone NAP, Maffei, Schimmenti & Certa PI); Avola (Bianca PI); Avola Cava dell'Amico (E. & A. Huet du Pavillon FI, RO); V.ne Caradonna di Canicattini Bagni, s.l. (FI) (specimens cited in Corsi et al. [21]); Cava of Cugno Sciurata, NE M. Climiti (Giardina et al. [39]). (Literature data obtained from Corsi et al. [21] and Giardina et al. [39])

Specimina visa of *U. rupestris*

In rupibus umbrosis vulcanicis, Militello, April, Todaro 992 (PAL 80667); Sortino Vallone del……Fontane 07/06/1829 (PAL 65512); Monti Climiti (Siracusa), 25/04/1988, *Scelsi F.* (CAT 29979); Valle dell'Anapo, Pantalica (Siracusa), 14/04/1988 (CAT 29981); Monti Climiti, Cava Monstringiano (Italia, Sicilia, Siracusa), 27/05/1989, *Bartolo G., Brullo S., Pulvirenti S., Scelsi F.* (CAT 29978); Monti Climiti, Cava Sorciaro (Siracusa), 23/03/1989, *Minissale P., Pavone P., Spampinato G.* (CAT 29980); Ferla (Siracusa), 01/06/1989, *Fichera G., Scelsi F.* (CAT 29982); Valle dell'Anapo (Sortino), 15/06/1990, *Raimondo et al.*, (PAL 69328); A' proximité du Vallone Calcarone, commune de Militello Val di Catania, province de Catane, Sicile, 12/05/1995, *Certa G. Scafidi G., Schimmenti E.*, (PAL 65513); Vallone Calcarone, Militello Val di Catania (CT), 12/05/1995, *G. Certa, G. Scafidi & E. Schimmenti* (PAL 88013); Valle dell'Anapo, C.da Isola Ardito (SR), 25/05/1995, *L. Gianguzzi*, (PAL 88012, 88480); Valle dell'Anapo, Siracusa, 29/05/2006, *Minissale P., Sciandrello S., Spampinato G. s.n.* (CAT 9520); Anapo, Siracusa (Escursione SIF), 2/04/2006, Sciandrello S., s.n. (CAT); Valle Anapo (Siracusa), 13/07/2006, Sciandrello S., s.n. (CAT); Monti Climiti (Siracusa), 25/04/2007, *Zimmitti A.* (CAT 30938); Anapo, Siracusa (Case Specchio), 25/06/2014, Sciandrello S., s.n. (CAT); Grotta Monello (Siracusa), 14/01/2016, Sciandrello S., s.n. (CAT); Grotta Monello (Siracusa), 07/04/2017, Giusso G., Sciandrello S., s.n. (CAT); Grotta Palombara (Siracusa), 17/04/2019, Sciandrello S., s.n. (CAT); Monti Climiti, Contrada Scrivilleri (Priolo Gargallo), 210 m s.l.m., 15/07/2021, *Sciandrello S., Minissale P., Tavilla G.*, s.n. (CAT).
